# The Nicotinamide/Streptozotocin Rodent Model of Type 2 Diabetes: Renal Pathophysiology and Redox Imbalance Features

**DOI:** 10.3390/biom12091225

**Published:** 2022-09-02

**Authors:** Liang-Jun Yan

**Affiliations:** Department of Pharmaceutical Sciences, College of Pharmacy, University of North Texas Health Science Center, Fort Worth, TX 76107, USA; liang-jun.yan@unthsc.edu

**Keywords:** nicotinamide, streptozotocin, diabetic nephropathy, diabetic kidney disease, type 2 diabetes

## Abstract

Diabetic nephropathy (DN) is a common complication of diabetes mellitus. While there has been a great advance in our understanding of the pathogenesis of DN, no effective managements of this chronic kidney disease are currently available. Therefore, continuing to elucidate the underlying biochemical and molecular mechanisms of DN remains a constant need. In this regard, animal models of diabetes are indispensable tools. This review article highlights a widely used rodent model of non-obese type 2 diabetes induced by nicotinamide (NA) and streptozotocin (STZ). The mechanism underlying diabetes induction by combining the two chemicals involves blunting the toxic effect of STZ by NA so that only a percentage of β cells are destroyed and the remaining viable β cells can still respond to glucose stimulation. This NA-STZ animal model, as a platform for the testing of numerous antidiabetic and renoprotective materials, is also discussed. In comparison with other type 2 diabetic animal models, such as high-fat-diet/STZ models and genetically engineered rodent models, the NA-STZ model is non-obese and is less time-consuming and less expensive to create. Given that this unique model mimics certain pathological features of human DN, this model should continue to find its applications in the field of diabetes research.

## 1. Introduction

Diabetic nephropathy (DN), also known as diabetic kidney disease (DKD) [1,2,3], is a severe complication of diabetes mellitus [4,5]. Approximately 30% of diabetic patients can develop DN [6,7,8], which is also a chronic kidney disease and can progress to end-stage renal failure [9,10,11]. The hallmarks of DN are kidney hypertrophy [12,13,14], mesangial cell proliferation and mesangial matrix accumulation [15,16,17], glomerulosclerosis, and persistent levels of proteinuria [18,19]. Despite the great advancement in our understanding of the pathogenesis of DN and numerous approaches that have been tested to slow down DN development and progression, no effective therapeutics are currently available for the treatment of DN. This is because the specific mechanisms underlying the pathogenesis of DN are yet to be fully elucidated. Therefore, there are unmet needs in treating or halting DN. In this regard, animal models of diabetes [20], be they genetically engineered or chemically or dietary induced, are indispensable tools in DN research.

**Figure 1:**
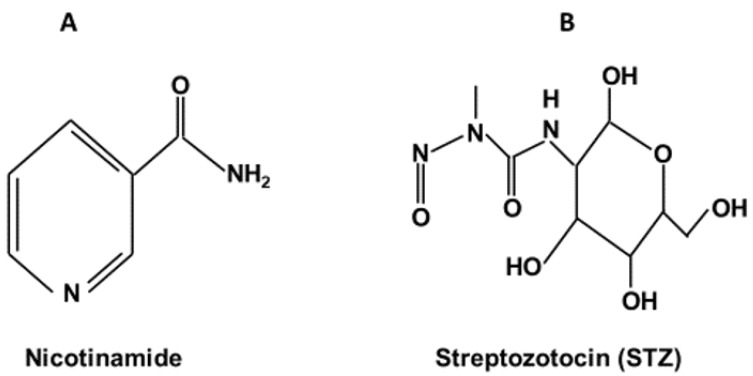
Chemical structures of nicotinamide and streptozotocin. (**A**): nicotinamide; (**B**): streptozotocin.

In this brief review, I focus on one particular type 2 diabetes animal model, which is created by using nicotinamide (NA, Figure 1A) and streptozotocin (STZ, Figure 1B) [21,22,23]. This non-obese type 2 diabetes animal model was initially established in rats [24] but has since been extended to include mice with modifications of the original protocol. In comparison with the high-fat-diet (HFD)-STZ-induced type 2 diabetes animal models [25,26,27,28] and genetically engineered diabetic animal models, such as db/db mice [29] and zsf1 obese rats [30], the NA/STZ model is time-saving and less expensive. Therefore, this model can be equally used as a platform for not only exploring the pathogenesis of DN but also screening and testing potential antidiabetic agents [31] or renoprotective compounds for their therapeutic effects [21].

## 2. Mechanisms Underlying NA/STZ Diabetes Induction

Masiello P. et al. initially developed this non-obese type 2 diabetes in rats in 1998 [24]. Since then, this model has been widely used to test a variety of antidiabetic materials for their beneficial effects on diabetes and diabetic complications, including DN. The establishment of this model takes advantage of the contradictory effects of the two chemicals on β cells as STZ is β cell cytotoxic while NAD is globally cytoprotective. Therefore, STZ-induced β cell damage can be blunted by nicotinamide [23]. Consequently, a certain percentage of β cells are viable and respond to glucose stimulation to release insulin [24]. It should be noted that the percentage of β cells that can survive really depends on the doses of the two chemicals. For a fixed dose of STZ, if the NA is too low, there will be no blunting effect from the NA and all β cells can be destroyed by STZ. On the other hand, if the NA is too high, the blunting or protective effects of the NA could be too high. In fact, the blunting or protective effects of the NA could reach 100% and no diabetes would be induced. For NA to play a protective role, NA is often given before STZ administration. Nonetheless, that NA is given shortly after STZ ingestion has also been reported in the literature [32,33,34,35]. In these cases, however, whether there are any differences between NA being given first or STZ being given first in diabetes induction and the severity of kidney injury has not been investigated. Notwithstanding, based on the observation that NA given immediately after STZ is equally protective [36], any difference should be minimal when the NA is administered right after STZ administration.

**Figure 2:**
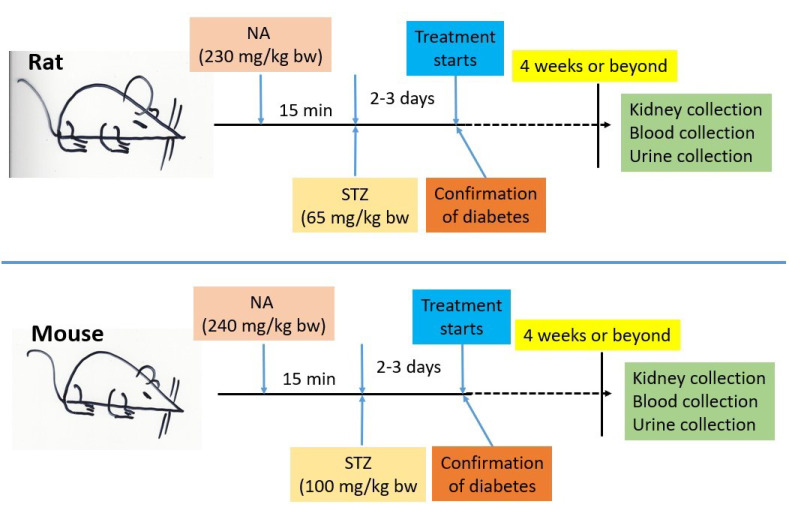
Diagrams showing representative flow charts of non-obese type 2 diabetes animal models induced by nicotinamide and streptozotocin. Renoprotective materials can be tested for their beneficial effects by this model, which is also outlined in the diagram. Note that for mice to be used as a model, more than one NA and STZ injection may be performed. Depending on the objective of a given study, the mouse model of NA-STZ diabetes induction may also involve HFD feeding for weeks before NA and STZ administrations (please see the text for a detailed discussion).

STZ is a nitrosourea compound that has a component similar to glucose (Figure 1B) [37]. Hence, STZ is also known as a glucose analog [37]. Because of this structural similarity to glucose, the STZ enters into β cells via the glucose transporter-2 (Glut2) [37] that is abundantly expressed on the β cell surface [38]. Once inside the β cells, the nitrosoamide moiety of STZ can attack DNA and causes DNA alkylation and is thus responsible for STZ genotoxicity and cytotoxicity [23]. STZ-caused DNA damage can activate poly (ADP-ribose) polymerase-1 (PARP-1) that can then repair damaged DNA using NAD+ as a substrate [39]. As a result, NAD+ could be potentially depleted by the activated PARP-1 [23], thereby leading to cell death. When NA is administered prior to STZ administration, the damaging effect of STZ is greatly mitigated. This mitigating effect is thought to be due to two establishments. One is that NA is a direct inhibitor of PARP-1 [23], the other is that NA is a precursor of NAD+ [23]. Hence, the STZ cytotoxic effects can be greatly blunted by NA and the blunting magnitude is known to be NA-concentration-dependent [23]. In rats, although different investigators would use a different dosage combination of NA and STZ, the initially established dosages of NA and STZ (230 mg/kg and 65 mg/kg, respectively) still seem to prevail in the literature (Figure 2), though the application of lower concentrations of NA and STZ has been reported. In mice, the ingested concentrations of the two chemicals also vary widely. Nevertheless, it should be noted that the concentration of STZ for mice can be higher than that for rats. It appears that the use of 240 mg/kg NA and 100 mg/kg STZ in a mouse model is a prevailing approach in the literature [40]. It should also be noted that when a mouse is used as a model, multiple daily injections of NA and STZ (up to a week) may be conducted [41]. Certain investigators have also reported using high-fat-diet (HFD) feeding followed by NA and STZ ingestions [40]. Regardless of whether rats or mice are being modeled, the key point is that a given investigator should stick to their own protocol of NA and STZ administrations, such as the dosages and routes of chemical ingestions [21,23], so that a reproducibility and data comparison could potentially be achieved. It should also be noted that the severity of the diabetic disorders depends on how long the animals are kept after diabetes induction by the two chemicals. In the absence of any interventions, diabetic disorders will progress, mimicking various stages of clinical practice in humans.

Additionally, based on a modified mouse model of type 2 diabetes induced by combining the HFD-NA-STZ treatments [42], investigations of DN created by HFD feeding in conjunction with NA and STZ administrations have also been reported [43,44]. It should be noted that when an HFD is involved, the creation of such a model would take longer than when only NA and STZ are used.

## 3. Renal Pathophysiology in this NA/STZ Animal Model

It has been reported that when diabetes was induced in mice by injection of 230 mg/kg NAD along with 50 mg/kg or 65 mg/kg STZ, the kidney organ index (kidney weight vs. body weight) for both STZ doses showed an increase when compared with that of the controls [45]. For a six-week duration of testing, urinary and serum parameters, such as creatinine, urea, and uric acid, were enhanced in the NA-STZ diabetic animals. In the presence of NAD, mice lived longer than those that received only STZ administration [45]. Such a result further demonstrates the blunting effects of NA on STZ cytotoxicity.

**Figure 3:**
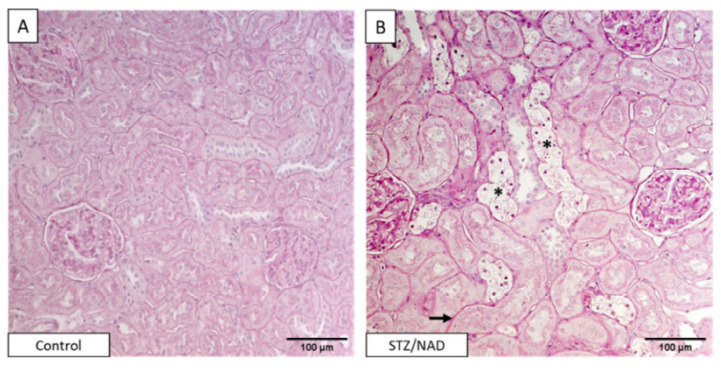
Histopathology staining of DN. Periodic acid–Schiff (PAS)-stained renal sections of a non-diabetic control rat (**A**) and STZ/NAD (**B**) diabetic rat at week 12. Indicated are tubular epithelial cell necrosis (asterisk), thickening of tubular basement membrane (arrow). This figure was reproduced from Corremans et al. [48].

**Figure 4:**
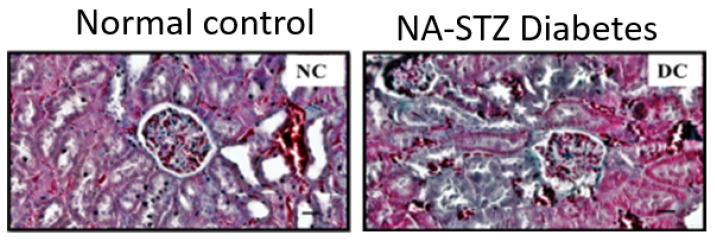
NA-STZ diabetic kidney histopathology stained by Masson trichrome. Kidney tissues were collected and processed for staining after 28 days of diabetes induction. This figure was reproduced from Arigela et al. [49].

It should be noted that while DN can be classified into five stages (Table 1), none of the NA-STZ-involved animal studies published so far have systematically addressed the five stages of diabetic kidney injury. Therefore, future studies on the progression of DN from stage 1 to stage 5 in the NA-STZ animal model need to be conducted. Moreover, numerous diabetic kidney injury biomarkers, such as those recently reported by Pelle et al. [46] and Natesan et al. [11], have also not been systematically and comprehensively evaluated in this NA-STZ animal model. Most studies use popular kidney injury parameters [47] such as blood urea nitrogen (BUN), serum cystatin C, creatinine, uric acid, or/and estimated glomerular flow rate (eGFR) for the evaluation of diabetic kidney injury after NA-STZ injections. The histopathological staining of kidney is also frequently used for the analysis of kidney injury in this NA-STZ animal model. Figure 3 and Figure 4 [48,49] show a typical staining of the kidney tissues by the periodic acid–Schiff and Masson trichrome, respectively. As can be observed from these histochemical stainings, the pathophysiological changes are obvious in the NA-STZ diabetic kidneys. Table 2 summarizes the renal pathophysiological measurements in the NA-STZ diabetic animal models in the absence of any interventions.

## 4. Application of this Model in DN Research

**Figure 5:**
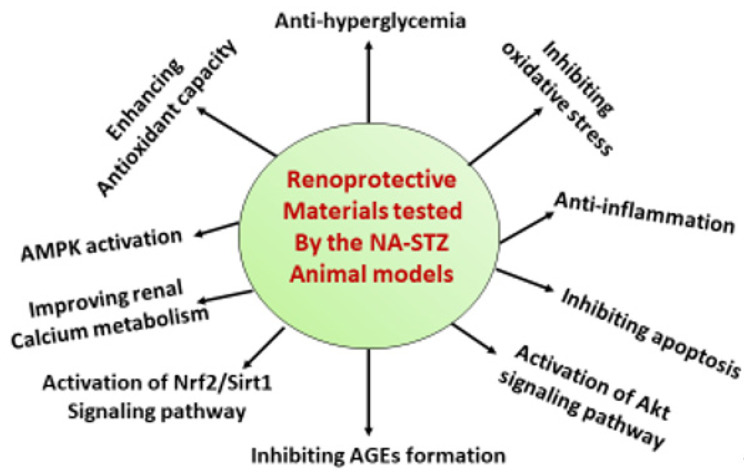
Diagram summarizing the representative renoprotective mechanisms of the materials listed in Table 3, using the NA-STZ non-obese type 2 diabetes animal models. AGEs stands for “advanced glycation end products”.

As mentioned above, this NA-STZ diabetes animal model is a non-obese type 2 diabetes model [23]. The pathogenesis of diabetes in this model may be different from that of HFD/STZ or genetically engineered models, such as the db/db mouse model and the zsf1 obese rat model [30,94,95,96]. Nevertheless, the NA-STZ model may provide a unique platform for the study of non-obese diabetes and diabetic complications [43,97]. With respect to DN research, this model has been widely used for testing the therapeutic effects of numerous antidiabetic or renoprotective materials (Table 3). Most of these materials are natural products derived from plants, such as herbs, trees, teas, and vegetables. Table 3 lists the representative materials that exhibit renoprotective effects in DN in the NA-STZ rodent model of type 2 diabetes. It should be noted that as oxidative stress has been established as one of the major mechanisms underlying DN, many of the listed materials in Table 3 thus have antioxidant properties. Figure 5 summarizes the major mechanisms of the renoprotective materials tested by this NA-STZ animal model. It should also be noted that the renoprotective effects of many of the tested materials are in a dose-dependent manner, such as reported in reference [98] and others.

## 5. Redox-Related Mechanisms That Remain to Be Elucidated in this NA-STZ Model

**Figure 6:**
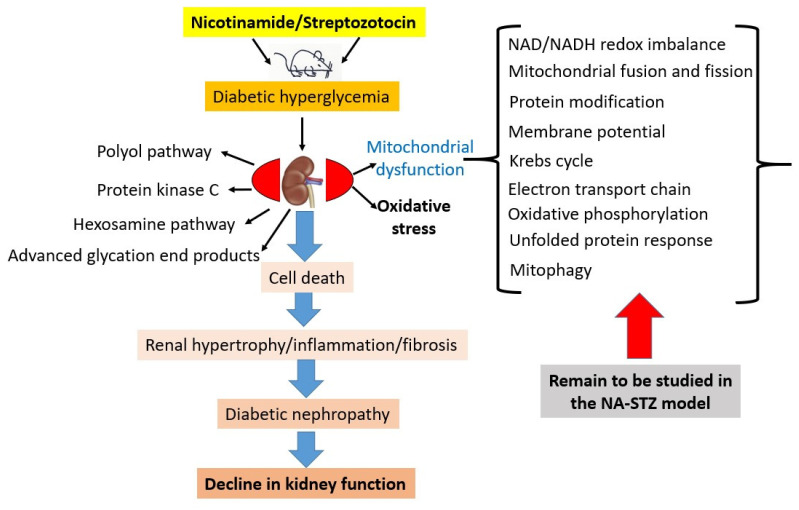
Potential mechanisms underlying diabetic nephropathy in the NA-STZ animal model. While common deleterious mechanisms operate in the kidney upon hyperglycemic challenge, those potential mitochondrial mechanisms underlying kidney injury remain to be elucidated (right side of the figure). These deleterious mechanisms would eventually converge on renal hypertrophy and renal fibrosis, leading to phenotype of diabetic nephropathy and kidney functional decline.

The non-tissue-specific mechanisms involved in cellular injury are thought to be implicated in the development of diabetic nephropathy [5,50]. These mechanisms, as shown in Figure 6, include the activation of the polyol pathway [105] and protein kinase C signaling, the hexosamine pathway, and the increased formation of the advanced glycation products [5,106]. However, what has been lacking is the underlying pathological mechanisms of DN in this unique NA-STZ model, in particular, redox signaling and the mitochondrial mechanisms of NA-STZ-induced DN. In fact, numerous aspects remain to be investigated in detail. These include mitochondrial redox imbalance [39]; sources of mitochondrial reactive oxygen species [107]; proteomics of mitochondrial protein oxidation [108,109]; mitochondrial abnormalities such as the derangement of mitochondrial metabolic pathways, including the Krebs cycle and electron transport chain [29]; fatty acid oxidation [110,111]; mitochondrial fusion and fission [112,113]; and mitophagy and the mitochondrial unfolded protein response [114,115,116,117,118,119] (Figure 6). The changes in redox signaling during the progression of DN in this animal model also remain to be comprehensively studied. Nephron segment-specific investigations of targeted genes [120,121] as well as the role of epigenetics [122,123] in this DN model also remain to be fully conducted. Delineating the mechanisms of these biological processes in the diabetic kidney may provide comprehensive insights into the underpinnings of DN. Additionally, this model could also provide a platform for testing the therapeutic effects of stem cells and gene therapy on DN [11]. For studying multiple kidney disease-causing risk factors, this model could also be combined with other kidney disease animal models, such as those induced by folic acid [47,124,125,126], cisplatin [127,128], cadmium [129,130,131], lipopolysaccharide [132,133], and hypoxia or ischemia reperfusion [134,135,136,137,138,139,140,141].

Finally, this NA-STZ diabetes animal model may also be used to evaluate any potential renoprotective effects of caloric restriction [142,143,144], intermittent caloric restriction [145,146], exercise [147,148,149,150], and ketone bodies [151,152,153,154], which all have been demonstrated to provide beneficial effects on the kidney in a variety of pathological conditions [155,156]. Indeed, the underlying mechanisms of renoprotection conferred by these approaches in this non-obese type 2 diabetes model remain to be comprehensively elucidated.

## 6. Summary

The NA-STZ induction of a type 2 diabetic animal model is a useful tool for both studying the mechanisms of DN and screening renoprotective materials for diabetic kidney disease. The model is less time-consuming and less expensive than that created by genetic engineering or high-fat-diet feeding. The establishment of this model is based on the fact that NA can partially protect pancreatic β cells against STZ cytotoxicity, leading to the incomplete destruction of β cells and thus development of non-insulin-dependent type 2 diabetes mellitus [21,23,24]. This unique animal model should continue to serve as a utility for studying the non-obese type 2 diabetes that is highly prevalent in East Asian diabetic patients [157].

## Figures and Tables

**Table 1:** Pathophysiological stages of diabetic nephropathy.

Stage 1: Glomerular basement membrane thickening, normal GFR *, no urinary albumin; high blood pressure is often observed
Stage 2: Mild to severe mesangial expansion, increased mesangial matrix, normal GFR
Stage 3: Damaged glomerular and increased albuminuria can be observed. This stage is also known as nodular sclerosis
Stage 4: Advanced stage of glomerulosclerosis
Stage 5: Complete kidney failure, GFR is well below 15 mL/min/1.73 m^2^

* GFR: glomerular flow rate. This table is adapted from Agarwal R. [50] and Natesan V. et al. [11].

**Table 2:** Renal pathophysiology in the NA-STZ rodent model.

Rodent Model (NA/STZ, mg/kg)	Analysis Time Point after NA/STZ Injections	Measured Renal Pathophysiology	Reference
Rat (200/55)	2 months	Increased serum Cre and proteinuria, advanced glomerulosclerosis	[51]
Rat (110/65)	4 weeks	Increased kidney index and BUN, decreased NAD and ATP contents in renal cells, increased oxidative damage	[52]
Rat (110/65)	6 weeks	Increased renal triglycerides, enlarged Bowman’s capsule, Congested glomeruli, elevated serum Cre and BUN	[53]
Rat (200/65)	2 weeks	Increased renal vitamin A and C	[54]
Rat (110/55)	4 weeks	Increased BUN and serum creatinine, increased renal Oxidative stress, and decreased renal antioxidants	[49]
Rat (230/65)	30 days	Increased levels of serum urea, uric acid, creatinine, and BUN	[55]
Rat (200/55)	21 days	Increased urinary α1-macroglobulin excretion, increased serum uric acid and BUN, and enlarged glomerular diameter	[56]
Rat (120/50)	4 weeks	Elevated serum fructosamine, increased serum creatinine and urea	[57]
Rat (100/50)	8 weeks	Increased urinary N-acetyl-β-D-glucosaminidase, urea uric acid, and Cre	[58]
Rat (110/65)	6 weeks	Increased serum Cre, BUN, and uric acid	[59]
Rat (110/65)	6 weeks	Increased kidney–body index, elevated levels of serum Cre, BUN, uric acid, and urinary protein	[60]
Mouse (180/60/HFD)	8 weeks	Increased serum Cre and kidney–body index	[61]
Rat (110/65)	4 weeks	Increased serum Cre and BUN	[62]
Rat (230/65)	45 days	Elevated levels of BUN, serum uric acid, and Cre	[63]
Rat (230/65)	8 weeks	Increased ratio of urinary albumin to urinary Cre	[64]
Rat (230/65)	12 weeks	Increased albuminuria and increased serum Cre	[65]
Rat (110/65)	35 days	Increase in histological tubular injury	[66]
Rat (230/65)	30 days	Increased levels of serum urea, uric acid, Cre, and BUN	[67]
Rat (110/45)	45 days	Multiple foci of hemorrhage, necrosis, and swelling tubules	[68]
Rat (120/40)	4 weeks	Increased BUN and serum Cre, increased kidney index and glomerular size	[69]
Mouse (120/60)	5 weeks	Increased levels of BUN, serum Cre, uric acid, and urea, elevated levels of urine protein	[70]
Rat (110/65)	40 days	Increased levels of urea, uric acid, and Cre in the sera	[71]
Rat (120/60)	5 weeks	Increased renal tubular vacuolation and tubular degeneration	[72]
Rat (120/60)	45 days	Increased levels of serum urea, uric acid, and Cre	[73]
Rat (85/65)	8 weeks	Increased serum glucose, urea, and Cre with albuminuria	[74]
Rat (110/55)	6 weeks	Increase in: Kim-1, serum Cre, BUN, uric acid, and urine albumin/Cre ratio	[75]
Rat (110/55)	6 weeks	Glomerular and tubular injuries observed histochemically	[76]
Rat (230/65)	12 weeks	Increased serum Cre and albumin to Cre ratio, glomerular and tubular injury observed histochemically	[48]
Rat (230/55)	6 weeks	Increased serum Cre and BUN with decreased urine Cre	[77]
Mouse (240/100/HFD)	8 weeks	Increased microphage infiltration in the kidney	[78]
Rat (230/65)	45 days	Increased levels of blood urea, uric acid, BUN, and Cre	[79]
Rat (110/55)	28 days	Decreased Cre clearance, increased BUN and uric acid, increased urine protein contents	[80]
Rat (110/65)	21 days	Decrased renal antioxidant power with increased renal oxidative damage	[81]
Rat (230/65)	12 weeks	Increased hemorrhage and neutrophils gathering in the kidney	[82]
Rat (110/50)	30 days	Decrease in Cre clearance, tubular lumen dilation, swelling of proximal tubular cells with tubular cell necrosis and intraluminal casts	[83]
Rat (100/60)	4 weeks	Increased kidney index, increased urine albumin, thickening of the basement membrane of renal tubule	[84]
Rat (110/45)	45 days	Increased levels of Cre and proteinuria, podocyte hypertrophy	[85]
Mouse (120/100)	4 weeks	Increased fibrotic deposition in the kidney	[86]
Rat (120/60)	60 days	Increased urine volume and urine albumin, increased serum uric acid	[87]
Rat (110/65)	4 weeks	Tubules with vacuolated cells, glomerulai exhibiting mesangial thickening	[88]
Rat (120/60)	28 days	Increased blood urea, glomerular enlargement, and sclerosis	[89]
Rat (110/65)	45 days	Increased fatty acid contents in the kidney	[90]
Rat (210/55)	8 weeks	Increased BUN and serum Cre with elevated proteinuria	[19]
Rat (110/50)	6 weeks	Increased serum Cre, uric acid, and proteinuria, decrease in creatinine clearance	[91]
Rat (110/55)	28 days	Increased BUN, serum creatinine, and uric acid with proteinuria	[92]
Rat (100/55)	28 days	Increased serum Cre and urea, glomerular architecture deranged	[93]

Abbreviations: BUN, blood urea nitrogen; Cre, creatinine; HFD, high fat diet.

**Table 3:** Renoprotective materials tested by the NA-STZ type 2 diabetes animal models.

Renoprotective Materials	Rodent Model (NA/STZ, mg/kg)	Mechanism	Reference
1,8 Cineole	Rat (200/55)	Glyoxalase-I induction	[51]
Abroma augusta L leaf	Rat (110/65)	Inhibiting oxidative stress	[52]
Acetate	Rat (110/65)	Suppressing xanthine oxidase activity	[53]
Abrus precatorius leaf	Rat (110/60)	Total antioxidant increase in kidney	[98]
Ascomycetes	Rat (200/65)	Inhibiting oxidative stress	[54]
Betanin	Rat (110/45)	Antioxidative damage	[99]
Bitter Gourd Honey	Rat (110/55)	Antioxidation, anti-inflammation	[49]
Bocopa monnieri	Rat (230/65)	Inhibiting AGEs formation	[55]
Brucea javanica seeds	Rat (100/60)	Inhibiting alpha-glucosidase	[100]
Cichorium intybus L seed	Rat (200/55)	Improving blood and urine parameters	[56]
Citrus reticulate fruit peel	Rat (120/50)	Antioxidative stress	[57]
Combretum micranthum	Rat (100/50)	Elevating SOD and catalase activities	[58]
CoQ-10/metformin	Rat (110/65)	Inhibiting oxidative stress	[59]
CoQ-10/sitagliptin	Rat (110/65)	Enhancing antioxidant system	[60]
Cordyceps militaris	Mouse (180/60)	Decreasing serum creatinine levels	[61]
Crocin	Rat (110/65)	Antioxidation	[62]
Curculigo orchiodies	Rat (230/65)	Antioxidation, anti-hyperlipidemia	[63]
Dapagliflozin	Rat (230/65)	Normalizing renal corpuscles histology	[64]
Dapagliflozin/irbesartan	Rat (230/65)	Inhibiting AGEs formation	[65]
Dietary flaxseed	Rat (110/65)	Antioxidative stress	[66]
Dillenia Indica L	Rat (230/65)	Inhibiting AGEs formation	[67]
Diosmin	Rat (110/45)	Inhibiting oxidative stress	[68]
Ellagic acid/pioglitazone	Rat (175/65)	Improving kidney function markers	[101]
Empagliflozin	Rat (120/40)	Decreasing BUN, creatinine, and oxidative stress	[69]
Eysenhardtia polystachya	Mouse (120/60)	Inhibiting glycation	[70]
Garlic extract	Rat (110/65)	Inhibiting oxidative stress	[71]
Grain amaranth	Rat (120/60)	Improving renal calcium metabolism	[72]
Glycosin	Rat (120/60)	Decreasing blood urea and creatinine	[73]
Hypericum perforatum	Rat (85/65)	Antioxidative stress	[74]
Lipoic acid	Rat (110/55)	Activating CSE/H_2_S pathway	[75,76]
L-NAME	Rat (230/65)	Increasing blood glucose	[48]
Manilkara zapota extract	Rat (120/60)	Reversing glomerulosclerosis	[102]
Metformin	Rat (230/55)	Decreasing BUN and serum creatinine	[77]
Myrciaria cauliflora	Mouse (240/100)	Inhibiting oxidative stress	[78]
Naringenin	Rat (120/60)	TRB3-FoxO1 downregulation	[97]
Oligo-fucoidan	HFD-Mouse (200/50)	Activation of Nrf2 and Sirt1	[41]
Paeonia emodi	Rat (230/65)	Inhibiting glycation end products	[79]
Phyllanthus niruri leaves	Rat (110/55)	Antioxidative stress	[80]
Pioglitazone	Rat (110/65)	Antioxidation	[81]
Pomegranate	Rat (120/60)	Antioxidative stress	[103]
Quercetin	Rat (230/65)	Anti-apoptosis	[82]
Resveratrol	Rat (110/50)	Attenuating oxidative stress	[83,104]
Rhinacanthins	Rat (100/60)	Inhibiting oxidative stress	[84]
S-allylcysteine	Rat (110/45)	Attenuating oxidative stress	[85]
SGLT2 inhibitors	Mouse (120/100)	AMPK activation	[86]
Silymarin	Rat (120/60)	Lowering serum creatinine and uric acid	[87]
Strawberry	Rat (110/65)	Enhancing kidney antioxidant defense	[88]
Syzygium calophyllifolium Rat	(120/60)	Enhancing kidney antioxidant defense	[89]
Tetrahydrocurcumin	Rat (110/65)	Preventing fatty acid changes in the kidney	[90]
Tetramethylpyrazine	Rat (210/55)	Akt signaling pathway activation	[19]
Vanillic acid	Rat (110/50)	Attenuating oxidative stress	[91]
Tilianin	Rat (110/55)	Nrf2 signaling pathway activation	[92]
Zanthoxylum			
Zanthoxyloides extract	Rat (100/55)	Improved kidney histology and biomarkers	[93]

Note: This table is not meant to be exhaustive and only shows the materials tested for their renoprotective effects. Therefore, antidiabetic materials screened using this model but not focusing on diabetic nephropathy are not included in this table. AGEs = advanced glycation end products.

## Data Availability

Not applicable.
